# OpenVac: An open-source low-cost fine vacuum sensor

**DOI:** 10.1016/j.ohx.2025.e00714

**Published:** 2025-10-08

**Authors:** Julius Bernd Zimmermann, Marcus Herbig

**Affiliations:** Department of Inorganic Chemistry, Technische Universität Bergakademie Freiberg, Leipziger Str. 29, 09599 Freiberg, Germany

**Keywords:** Vacuum, Sensor, Measurement, Low-cost, DIY, Mobile

## Abstract

When it comes to moisture- or air-sensitive chemicals, often the Schlenk technique is used to achieve safe and reliable results. In this article, we present an open-source and low-cost microcontroller-based alternative for vacuum sensors for this application. In Schlenk technique, the atmosphere in the reaction apparatus is replaced with an inert gas like argon or nitrogen. To do so, the apparatus first needs to be evacuated. To monitor the pressure level, vacuum sensors are used. Commercially available vacuum sensors typically are expensive and hard or even impossible to repair. Thus, we like to present our open-source and low-cost alternative, which uses a Pirani-type sensor and a Wheatstone bridge. The cross-voltage in this bridge circuit is amplified by an OpAmp and converted into a digital signal through a 12-bit ADC. The processing of this data is achieved through an ATtiny84, which also controls a 0.91-inch OLED display to show the current pressure. The device is battery powered and optimized for long runtime.

## Graphical abstract

**Figure:**
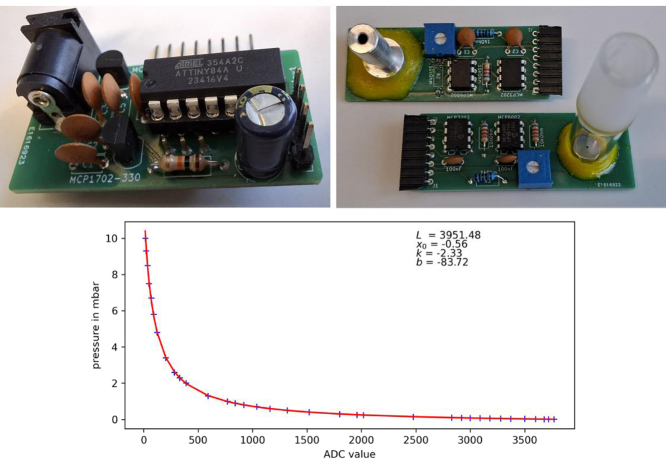

## Specifications table

**Table:** 

Hardware name	An open-source low-cost fine vacuum sensor

Subject area	
	•*Engineering and material science*
	•*Chemistry and biochemistry*
	•Educational tools and open-source alternatives to existing infrastructure
	•*General*

Hardware type	
	•Measuring physical properties and in-lab sensors
	•*Field measurements and sensors*

Closest commercial analog	
	•VACUUBRAND DCP + VSP 3000 (1330 EUR)
	•VACUUBRAND Vacuu View extended (1010 EUR)
	•WELCH Vacuum VMpro 2 (1136 EUR)
	•WELCH Ilmvac PIZA 111 (817 EUR)

Open-source license	CC-BY 4.0

Cost of hardware	approx. 70 EUR

Source file repository	https://doi.org/10.5281/zenodo.15728968

	

## 1 Hardware in context

Pressure is a key physical quantity that plays an important role in both industry and research. Various techniques are used to measure pressure, depending on the expected measuring range and the medium in question. In general, pressure can be measured relative to a known pressure or “absolute” (= relative to perfect vacuum conditions). Pressure sensors are often integrated into systems for monitoring environmental parameters, e.g., [1], [2]. Among other things, air pressure is a decisive factor in weather forecasting and has a typical measuring range of around 1 bar. Pressure also serves as a measured and controlled variable in the monitoring of industrial plants [3]. In medicine, there are several applications for increased or decreased pressure, e.g., negative pressure wound therapy [4]. In the field of research, pressure sensors (overpressure) are used, for example, for ocean profiling [5]. For many fundamental research applications or modern analytical techniques, a very low absolute pressure is needed. Even if this pressure is not a perfect vacuum, this pressure range is called a “vacuum” in several fields.

**Vacuum**

Vacuum or reduced pressure plays an important role in many modern technological applications. In addition to a way of creating this vacuum, such as through vacuum pumps, it is equally significant to be able to assess the quality of this vacuum. The classification is based on different pressure ranges and is referred to as rough, fine, high, ultra-high, and extremely high vacuum [6]. Each pressure range is suitable for different applications. While rough vacuum is used, for example, in the kitchen for cooking food or degassing liquids, high vacuum is found, for example, in the interior of electron tubes. High vacuum is used in electron microscopes or mass spectrometry devices. Ultra-high vacuum and extremely high vacuum are used when even longer mean-free paths are required, such as in gravitational wave detectors or particle accelerators. [7] Vacuum is also needed in chemical laboratories, whether for distillation, filtration, sublimation, or drying products. Especially in vacuum distillation, it is necessary that the vacuum is not only stable but can also be read as accurately as possible.

**Measuring the reduced pressure**

To measure the residual atmospheric pressure in low-pressure applications, special sensors are used. These sensors can be categorized into analog and electrical pressure sensors. Analog sensors are, for example, U-tube manometers (mercury column) or Bourdon pressure gauges. Advantages of analog measurement devices include simplicity and relatively low cost compared with the electric sensors. Typical pressure ranges go up to -100 kPa (< 1 mbar) but show a relatively high inaccuracy of 10 mbar. For example, a mercury-filled U-tube manometer can only measure down to 1 Torr (= 1 mmHg = 1.33322 mbar) accurately. The McLeod gauge can measure down to (< 10 × 10^-5^ Torr, but it is slow, not suitable for continuous measurements, and uses toxic mercury [8]. Because of the low accuracy and the measurement range, these analog devices are suitable for rough detection of vacuum but fail when precise values are needed or measurements are taken outside the rough vacuum range.

Electrical devices can be miniaturized [9]. The devices can be grouped by the measurement principle. The most common principles are:

• **Ceramic Diaphragm Pressure Sensor:** A change in pressure causes expansion or compression of a metal or ceramic, thereby increasing (expansion) or decreasing (compression) the resistance. Further differentiation is made between resistive or piezoresistive materials. In piezoresistive materials, the change in resistance due to the piezoelectric effect during compression and expansion is further amplified. Resistance measurement is done using a Wheatstone bridge. The pressure range is normally from the atmosphere to 1 × 10^-1^ mbar (rough vacuum).
• **Pirani-type Sensor:** Based on the pressure dependence of the thermal conductivity of gases, a thin measuring wire is heated electrically by using a heating voltage. The heat is transferred to the surroundings by irradiation as well as convection. The convection part of the heat flow strongly depends on the pressure of the surrounding gas. The resistance of the wire also changes with temperature (resistance temperature detectors), so that at high pressures, better heat transport (convection) occurs, leading to a lower temperature of the wire and thus a lower resistance. Resistance measurement is done using a Wheatstone bridge. Depending on the amplification of the cross-voltage and the ADC used, a low deviation can be achieved. The pressure range is normally from atmosphere to 5 × 10^-4^ mbar(rough vacuum to fine vacuum).
• **Ionization vacuum gauges (cold cathode by Penning, hot cathode by Bayard-Alpert)** are mainly intended for pressures in the high to ultra-high vacuum range. They are based on the principle that gas atoms are ionized and can thus be detected as an ion current. The number of ionized gas atoms, and thus the ion current, is proportional to the prevailing pressure. There are different designs, such as those by Penning or Bayard-Alpert, which can reach down to 1 mbar as the upper limit. As the lower limit, down to 1 × 10^-12^ mbar (XHV) can be achieved. The upper limit is determined either by the occurrence of glow discharge or the burnout of the hot cathode.

When it comes to moisture- or air-sensitive chemicals, the Schlenk technique is used to achieve safe and reliable results [10]. Therefore, the air in the reaction apparatus is replaced with an inert gas like argon or nitrogen. To do so, the apparatus needs to be evacuated at first. Diaphragm pumps and oil-lubricated rotary vane pumps are used, generating a pressure range from rough to fine vacuum for this application. To monitor the pressure level, vacuum sensors are used. The target pressure range is 1 mbar to 1 × 10^-4^ mbar. Therefore, Pirani-type sensors are most suitable for this application. Major manufacturers for measuring devices for this application include VACUUBRAND and WELCH, and formerly Ilmvac (now also Welch). These vacuum meters operating in the range from atmosphere to fine vacuum have a price range of 1000 to 1300 EUR and are often costly and hard or impossible to repair. Inexpensive devices from Ilmvac, costing around 800 EUR, are no longer readily available today. Thus, we like to present our open-source and low-cost alternative, which uses a Pirani-type sensor.

There are some other projects using pressure sensors with microcontrollers, e.g., [2], [5], [11], [12], but there is no device for use in Schlenk technique so far.

## 2 Hardware description

In the fume hood and the chemical apparatus, corrosive gases may be present. Therefore, corrosion must be expected. To keep costs as low as possible, the hardware is divided into two parts: (1) the power and display board and (2) the sensor board. Both boards are connected by pin headers (male/female) and may be replaced separately when broken.

The sensor board is based on the Pirani-type sensor HVS04 from Heimann Sensor. As described in the datasheet [13], the part itself consists of two resistors, one resistor with a constant resistance $R_{k}$ (Pin 2) of 1 kΩ and one temperature-dependent resistor $R_{p}$ (Pin 3) which also has a resistance of 1 kΩ in the unheated state. To complete the Wheatstone bridge, the following resistors have been chosen. One resistor $R_{f}$ (R3) with a resistance 1 kΩ as a second constant resistor and one potentiometer RV1 with a maximum of 2 kΩ to balance the bridge and compensate for any minor errors in the other resistors.

(1) $$\frac{U_{p}}{U_{0}}=\frac{R_{p}+\Delta R_{p}}{R_{p}+\Delta R_{p}+R_{k}}-\frac{RV1}{RV1+R3}$$

The measurement is performed based on the balanced bridge. Due to the reduced pressure, the heat-loss on Rp is reduced, which leads to an increasing temperature, which then leads to an increasing electrical resistance ($\Delta R_{p}$). The Wheatstone bridge is then unbalanced, which can be seen in a potential difference ($\Delta U_{p}$) between the two midpoints. This potential difference is proportional to the change in resistance and thus also to the change in pressure, as seen in Eq. (1). With a bridge ratio $k=\frac{R_{p}}{R_{k}}=\frac{RV1}{R_{3}}$ of about 1 in the balanced state, the Wheatstone bridge is also operated at its maximum sensitivity.

**Fig. 1:**
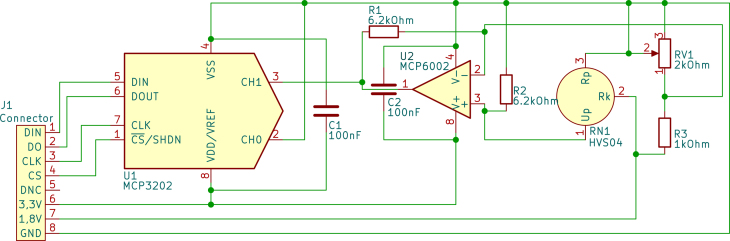
Schematics of the sensor board.

The sensor board schematics are shown in Fig. 1. The Wheatstone bridge is powered with 1.8 V. When applying pressure to the sensor, a maximum of about 360 mV of cross-voltage is reached. The cross-voltage is amplified using an MCP6002 OpAmp, and the amplified analog voltage is converted to a digital signal using an MCP3202 ADC. The digital signal is sent via SPI to the power part of the hardware.

**Fig. 2:**
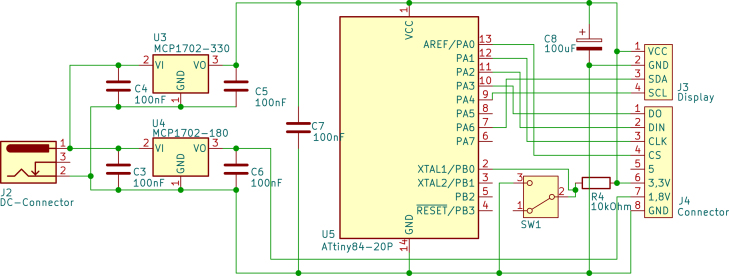
Schematics of the power and control board.

The power board (see Fig. 2) consists of two voltage regulators that produce 3.3 V for the digital part of the circuit and 1.8 V for the Wheatstone bridge of the sensor part. An ATtiny84-20P in this circuit board controls the sensor and the display. It gets the digital readout of the sensor board via SPI, calculates the pressure via a calibration (see below), and sends the information for displaying the pressure to the connected display via I${}^{2}$C.

The built-in switch allows for toggling between two modes during measurement. In measurement mode, only the converted pressure value is displayed, while in calibration mode, both the current ADC values and the averaged values from oversampling are shown. This allows for precise calibration and easier error detection in case of malfunction.

Power management is always a big topic when it comes to battery-powered devices. Therefore, we also minimized power consumption and maintained functionality. We achieved this by including the sleep function in the software so that the ATtiny84 fulfills its tasks and goes to sleep afterward. Together with the display and the periphery, the whole setup peaks at around 8.4 mA while awake. The ATtiny84 alone consumes about 3.2 mA, running on 3.3 V and 8 MHz. When put into sleep mode, this can be lowered to 3 µA so that the entire system only consumes about 4.98 mA. To minimize the time awake, it is convenient to run the ATtiny84 at 8 MHz at this time, although this consumes more power than running at a lower frequency.

**Fig. 3:**
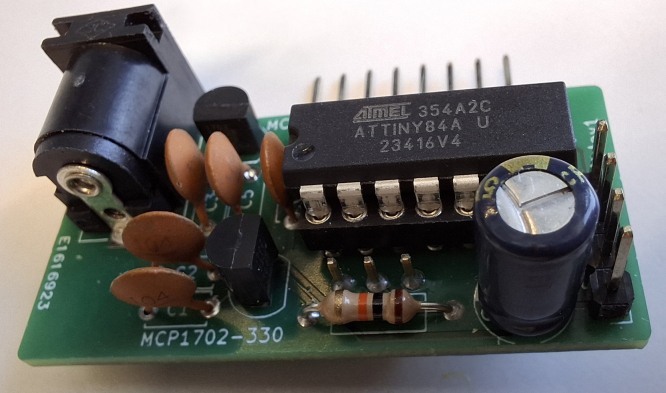
Photo of the power board with the inserted ATtiny84.

A housing is provided from a 3D-printed model (see Fig. 5). The connection of the sensor to the apparatus can be made in different ways, e.g., with a hose or a glass joint fixated on the board over the sensor with some epoxy resin. A photo of both types can be found in Fig. 3

Modification options exist for the power supply. We have limited ourselves to rechargeable 9 V block batteries due to their compact form and relatively high capacity (approximately 500 mA h). However, it is also possible to use other battery types as long as at least the required 3.3 V is achieved. Therefore, three AAA batteries or series-connected button cells could also be used. Additionally, wired power supply via USB (5 V) or the use of power banks is possible.

Furthermore, other display models can be used as well. We have used the most cost-effective model available, an OLED display with a screen diagonal of 0.91 inches (2.31 cm). Other models with higher pixel counts could be used, as well as displays with an SPI interface. The change to SPI-interfaced displays would also have the benefit of interfacing both ADC and display through the same communication protocol. As a result, only 5 instead of 6 pins on the ATtiny84 would be used. So it would be suitable to even use a smaller type of ATtiny, like the ATtiny85. This would result in saving space on the PCB. Another option would be to step away from OLED and use an LCD like the LCD1601. The benefit would be that if you power off the backlight of these displays, the power consumption could be minimized (< 1 mA) to further reduce the power consumption of the device. Although the readability of the display would be dependent on external lighting.

It would also be possible to improve accuracy by using ADCs with higher bit counts (16 or 24 bits), although the circuits may need to be optimized for noise in that case.

This hardware is designed for research labs using reduced pressure applications in the range of rough to fine vacuum, e.g., chemical labs but can also be interesting for other researchers due to the facts that:

• It is a low cost alternative to commercially available sensors, making it more accessible for labs with a limited budget or educational institutions.
• It can be used for applications like vacuum distillation, filtration, sublimation, vacuum drying or degassing of liquids.
• It is optimized for use with the Schlenk technique and corrosive atmospheres since it is divided into two parts making a cheap replacement of broken parts possible

## 3 Design files summary

**Table:** 

Design filename	File type	Open source license	Location of the file

pcb.scad	CAD file	CC-BY 4.0	Zenodo[14]
Case.scad	CAD file	CC-BY 4.0	Zenodo[14]
Case_slices.scad	CAD file	CC-BY 4.0	Zenodo[14]
edges.scad	CAD file	CC-BY 4.0	Zenodo[14]
Case_glass.scad	CAD file	CC-BY 4.0	Zenodo[14]
Case_glass_slices.scad	CAD file	CC-BY 4.0	Zenodo[14]
edges_glass.scad	CAD file	CC-BY 4.0	Zenodo[14]
Case_parts.stl	STL file	CC-BY 4.0	Zenodo[14]
Case_parts_glass.stl	STL file	CC-BY 4.0	Zenodo[14]
OpenVac_firmware.ino	Arduino sketch	CC-BY 4.0	Zenodo[14]
Calibration.py	Python file	CC-BY 4.0	Zenodo[14]
power_board.kicad_pcb	KiCAD PCB	CC-BY 4.0	Zenodo[14]
power_board.kicad_pro	KiCAD project	CC-BY 4.0	Zenodo[14]
power_board.kicad_sch	KiCAD Schematic	CC-BY 4.0	Zenodo[14]
sensor_board.kicad_pcb	KiCAD PCB	CC-BY 4.0	Zenodo[14]
sensor_board.kicad_pro	KiCAD project	CC-BY 4.0	Zenodo[14]
sensor_board.kicad_sch	KiCAD Schematic	CC-BY 4.0	Zenodo[14]

• pcb.scad: supporting CAD file with various models of the sensor and its components
• Case.scad: CAD file with a 3D model of the hose sensor housing
• Case_slices.scad: CAD file with slices of the hose sensor housing
• edges.scad: supporting CAD file with the edges for the hose sensor housing, which are included in the case slices
• Case_glass.scad: CAD file with a 3D model of the glass joint sensor housing
• Case_glass_slices.scad: CAD file with slices of the glass joint sensor housing
• edges_glass.scad: supporting CAD file with the edges for the glass joint sensor housing, which are included in the case slices
• Case_parts.stl: Stl file with the four parts of the hose sensor housing, ready for 3D printing
• Case_parts_glass.stl: Stl file with the four parts of the glass joint sensor housing, ready for 3D printing
• OpenVac_firmware.ino: Arduino sketch with the firmware for the sensor. Burn it to the ATtiny84 using the ArduinoIDE
• Calibration.py: Python script used for the calibration
• power_board.kicad_pcb: KICAD PCB file of the power board
• power_board.kicad_pro: KICAD Project file of the power board
• power_board.kicad_sch: KICAD Schematics of the power board
• sensor_board.kicad_pcb: KICAD PCB file of the sensor board
• sensor_board.kicad_pro: KICAD Project file of the sensor board
• sensor_board.kicad_sch: KICAD Schematics of the sensor board

## 4 Bill of materials summary

**Table:** 

Designator	Component	Number	Cost per unit - currency	Total cost - currency	Source of materials	Material type

C1–C7	Capacitor 100 nF	7	0.03EUR	0.21EUR	reichelt.de	Ceramic
C8	Capacitor 100 µF	1	0.28EUR	0.28EUR	reichelt.de	Polymer/Metal
J1	8 Pin Connector	1	3.70EUR	3.70EUR	reichelt.de	Polymer/Metal
J2	DC-Connector	1	2.10EUR	2.10EUR	reichelt.de	Polymer/Metal
J3	4 Pin Header	1	0.60EUR	0.60EUR	reichelt.de	Polymer/Metal
J4	8 Pin Header	1	0.30EUR	0.30EUR	reichelt.de	Polymer/Metal
R1, R2	Resistor 6.2 kΩ	2	0.04EUR	0.07EUR	reichelt.de	Metal
R3	Resistor 1 kΩ	1	0.035EUR	0.035EUR	reichelt.de	Metal
R4	Resistor 10 kΩ	1	0.038EUR	0.038EUR	reichelt.de	Inorganic
RN1	Sensor HVS04	1	23.20EUR	23.20EUR	heimannsensor.com	Metal
RV1	Potentiometer 2 kΩ	1	0.88EUR	0.88EUR	reichelt.de	Polymer/Metal
SW1	Switch	1	0.63EUR	0.63EUR	reichelt.de	Polymer/Metal
U1	ADC MCP3202	1	2.98EUR	2.98EUR	reichelt.de	Polymer/Metal
U2	OpAmp MCP6002	1	0.45EUR	0.45EUR	reichelt.de	Polymer/Metal
U3	LDO MCP1702-330	1	0.70EUR	0.70EUR	reichelt.de	Polymer/Metal
U4	LDO MCP1702-180	1	0.99EUR	0.99EUR	reichelt.de	Polymer/Metal
U5	ATtiny84-20P	1	2.10EUR	2.10EUR	reichelt.de	Polymer/Metal
	14 Pin IC Socket	1	0.25EUR	0.25EUR	reichelt.de	Polymer/Meta
Display	0.91inch OLED Display module	1	5.90EUR	4.94EUR	welectron.de	non-specific
DC Adapter		1	1.048EUR	1.048EUR	conrad.de	non-specific
Battery	9 VBattery, rechargeable	1	9.745EUR	9.745EUR	reichelt.de	non-specific
PCB	PCB sensor	1	4.58EUR	4.58EUR	eurocircuits.de	non-specific
PCB	PCB power	1	5.76EUR	5.76EUR	eurocircuits.de	non-specific
			TOTAL	66.546EUR		

						

Additional needed parts for programming and for the joint (may be needed):

**Table:** 

Component	Number	Cost per unit - currency	Total cost - currency	Source of materials

Arduino	1	9.66EUR	9.66EUR	reichelt.de
Breadboard	1	2.40EUR	2.40EUR	reichelt.de
Wires	1	2.10EUR	2.10EUR	reichelt.de
Glass joint blank	1	3.04EUR	3.04EUR	Rettberg
Epoxy resin	1	10.08EUR	10.08EUR	reichelt.de
		TOTAL	27.28EUR	

				

## 5 Build instructions

The build instructions are given below. For the most accurate results, we highly recommend doing your own individual calibration. But it is also possible to use the standard calibration, which is included in the firmware (see Fig. 11). In that case you can skip points 9–12. In the case that you want to use the device without a housing, you do not have access to 3D-printing or you want to use your own housing, you can also skip points 14–20.

**Fig. 4:**
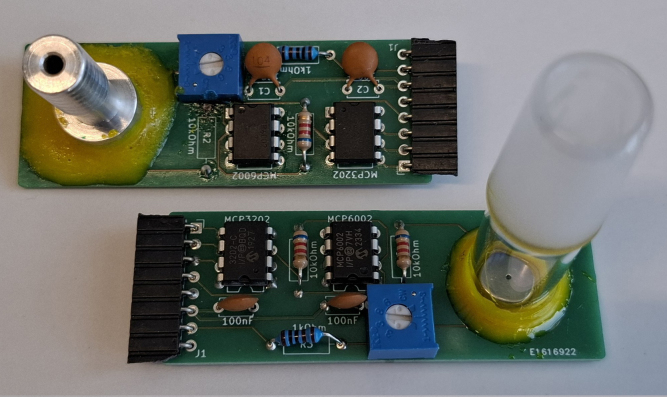
Photo of the sensor board with both types of connectors to the Schlenk line.

1. Solder all components (see BOM) on the right positions on the PCBs. Keep in mind that the Pin Header J4 differs depending on which gas connection you are planning to use. When using a glass joint it is recommended to use a straight pin header and mount it facing upwards. If your are planning to use a hose it is more convenient to use a bended 8 pin header and mount it facing downwards as seen in Fig. 4 or 6.

**Fig. 5:**
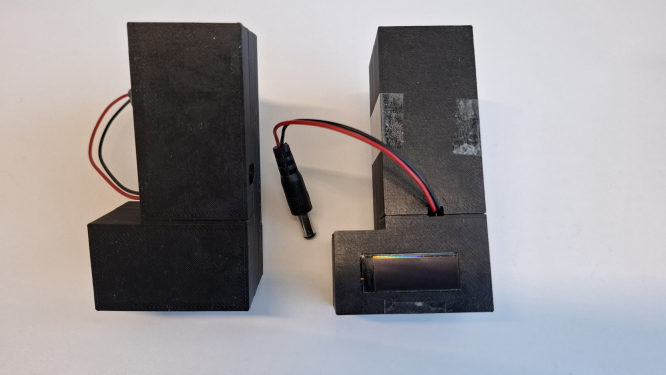
Photo of the 3D printed housing.

**Fig. 6:**
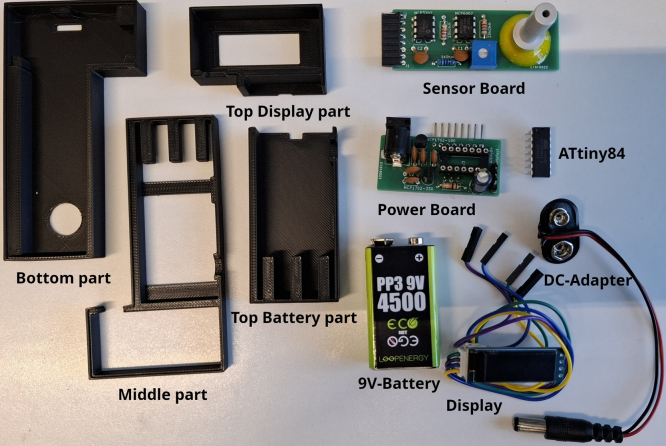
All parts needed for the assembly of the device.
2. Attach the gas connector (hose or glass joint) to the sensor on the sensor board (see Fig. 3) and fix it using epoxy resin.
3. Connect the power board and the display as shown in Fig. 7.

**Fig. 7:**
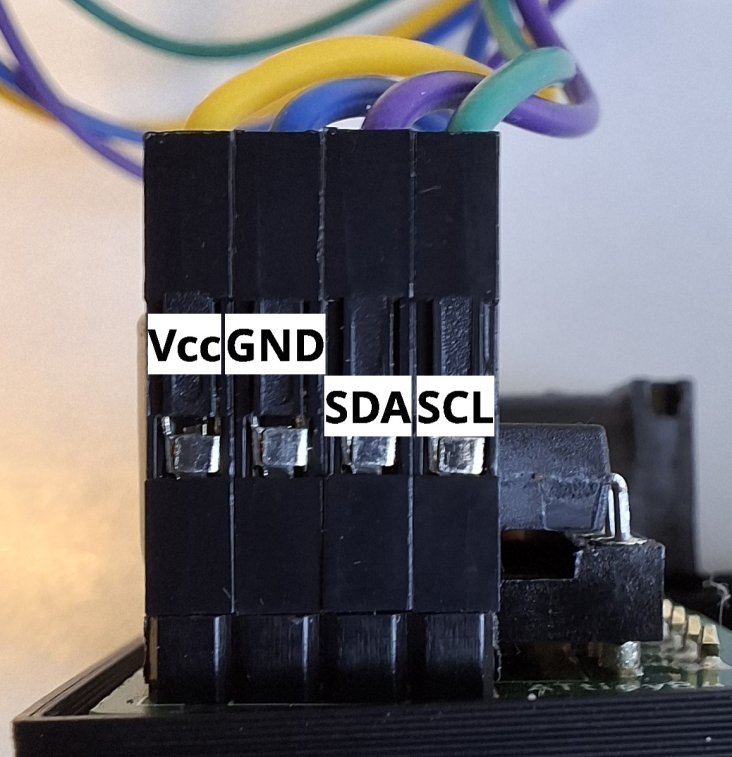
Connection of the power board and the display. Vcc: yellow; GND: blue; SDA: violet; SCL: green.
4. Burn the firmware with the standard calibration (*OpenVac_firmware.ino*) to the ATtiny84 for example by using the procedure given under “Programming the ATtiny”.
5. Insert the ATtiny84 in the socket on the power board as shown in Fig. 4.
6. Connect the sensor and the power board. Turn on the device by connecting to the power source of choice.
7. Make sure the switch is in calibration mode.
8. Calibrate by adjusting the potentiometer RV1 on the sensor board until the readout of the bridge is slightly above the minimum value (around 10) at around 100 mbar. This necessary to ensure that the Wheatstone bridge is operated at its maximum sensitivity within the measuring range.
9. Lower but constant pressure values have to be applied to the sensor. The pressure is read by another calibrated sensor in parallel to the one that needs to be calibrated (see Fig. 8). The readout is noted in connection with the current pressure.

**Fig. 8:**
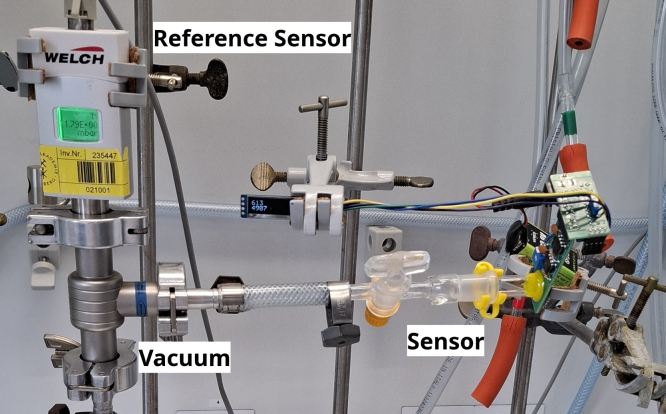
Apparatus for calibration.

**Fig. 11:**
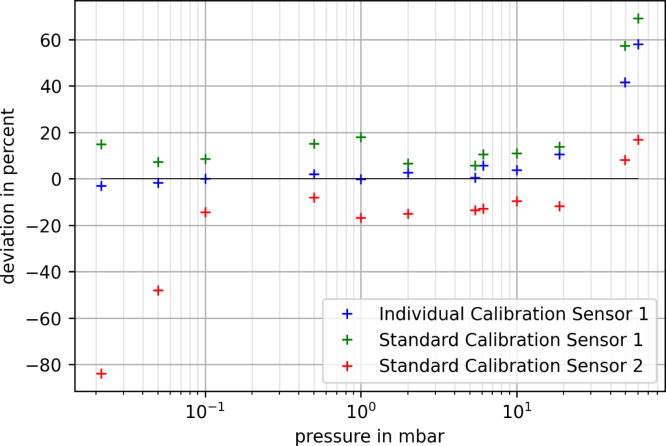
Relative deviation of the pressure values of different calibrations from the values of a commercial device.
10. For the calibration function, the pressure-readout values are fitted to the sigmoid function shown in Eq. (2) using the Python script *Calibration.py*.
  (2) $$readout\left(p\right)=\frac{L}{1+e^{-k\left(p-x_{0}\right)}}+b$$
  The sigmoid function is used since the datasheet [13] of the sensors shows a U(p) diagram with a sigmoid shape. The parameters L and $x_{0}$ define the upper and lower limits of the sigmoid function. L is the upper limit, which corresponds to the maximum voltage at the lowest pressure (up to 400 mV from the sensor [13], amplified by the op-amp). $x_{0}$ is the lower limit and should be near 0. The parameter b corrects an offset error of the measurement system. The most important parameter is k, which defines the sharpness of the bend of the curve. This bend is in the pressure range from 10 to 0 mbar, so it is the most important range for this application.
11. Power off the device by disconnecting the power source and remove the ATtiny from the power board.
12. Update the calibration parameters in the firmware to the new ones you determined, and repeat steps 4–6.
13. The device itself is now ready for operation.
14. Print the housing parts corresponding to your gas connection (hose: *Case_parts.stl*, glass joint: *Case_parts_glass.stl*) using a 3D printer.
15. Power off the device by disconnecting the power source.
16. Put the device in the bottom L-shaped part. Make sure that the gas connector and the DC connector sit in the right spot.
17. Add the middle part (also L-shaped).
18. Put the display in its gap and fixate it with some tape or hot glue.
19. Connect the battery and the DC adapter and insert it above the sensor part, making sure that the USB-C port aligns with the corresponding gap.
20. Close the housing by putting the two upper parts onto the device and fixate all with some tape.

**Manufacturing and Fixing the Gas Connection**

In this section, we are going to report first on how we manufactured the different gas connectors and, in the second part, how the gas connectors (glass joint or hose) are connected to the sensor board.

*Glass joint:*

The glass joint is the easier way to secure a good connection to the vacuum because glass joint blanks are widely commercially available and are easy to process. The NS 14.5 [15] blank just has to be cut to the desired length, e.g., 6 cm using a glass cutter, and then it can be attached to the sensor board. Furthermore, glass joints are a widely used connection type in the chemical laboratory, which makes it easier to connect the vacuum sensor to a Schlenk line or another type of apparatus.

*Metal Hose:*

A metal hose is the other type of gas connector we used to ensure a safe connection from the sensor board to the vacuum. Although it is more costly to use a metal hose, it also brings some advantages with it, like the higher durability due to metal as a material as well as a more flexible application because it is not dependent on a fitting glass joint on the apparatus. We manufactured the metal hose from aluminum using a lathe. The exact dimensions are highly dependent on the properties of the application. We manufactured them with an outer diameter of 8 mm and an inner diameter of 2 mm over a length of 22 mm and an outer diameter of 13 mm and an inner diameter of 10 mm over a length of 9 mm to leave enough space for the sensor part.

Fixing the gas connector to the sensor board:

1. Mix the two components of the epoxy resin
2. Put a circle of epoxy resin around the mounted sensor RN1
3. Press your gas connector of choice onto the epoxy resin ring and ensure that it is aligned straight.
4. Add more of the epoxy resin around the gas connector
5. Leave it to dry completely for about 24 h

To fix the gas connector to the sensor board, we used a type of two-component epoxy resin. Thus, we achieved a good connection between the board and the connector. To test this, we performed a leak test by closing the glass valve on the calibration apparatus (see Fig. 8) and tracking the pressure over time. The results are provided in the section Validation and Characterization.

**Programming the ATtiny**

Here, we want to show the procedure we used for programming the ATtiny. Although there are various ways to do so, we chose the path using an Arduino Nano and the Arduino IDE including the ATtiny Core Board Manager by Spence Code (https://github.com/SpenceKonde/ATTinycore). You can also use other methods for programming, but keep in mind that other hardware or even code changes may be needed.

1. Installing the right Board Manager (only done once):
  (a) Make sure you have installed the ArduinoIDE.
  (b) Navigate to File →Settings and click on “Additional Board Manager” URLs.
  (c) Insert “https://descartes.net/package_drazzy.com_index.json” and close by clicking on “OK”.
  (d) Navigate to Tools →Board →Board Manager, search for “attiny”and install *ATTinycore by Spence Konde*.
2. Setting up the Arduino (may only be done once):
  (a) Connect your Arduino to your PC by USB.
  (b) Navigate to File →Examples →ArduinoISP.
  (c) Choose the right port and upload the ArduinoISP sketch to your Arduino.
3. Programming the ATtiny:
  (a) Connect your Arduino and your ATtiny through a breadboard and some wires as shown in Table 1.

**Table 1:** Arduino ATtiny connection.

Function	Arduino pin	ATtiny pin
RESET	10	4 (PB3)
MOSI	11	7 (PA6)
MISO	12	8 (PA5)
SCK	13	9 (PA4)
GND	GND	14 (GND)
5V	5V	1 (VCC)
  (b) Open *OpenVac_firmware.ino* [14] with the ArduinoIDE.
  (c) Navigate to Tools →Board →ATTinycore →ATtiny24/44/84(a) (no Bootloader).
  (d) Choose the right settings: Chip: ATtiny84(a), Clock Source: 8 MHz (internal), Pin mapping: Clockwise, millis()/micros(): Disabled, BOD Level: BOD disabled.
  (e) Choose the right port.
  (f) Click on Tools →Burn Bootloader.
  (g) Upload the firmware by pressing Ctrl + Shift + u or by clicking Sketch →Upload with programmer.
  (h) Wait until the upload is finished and remove the ATtiny

## 6 Operation instructions

**Fig. 9:**
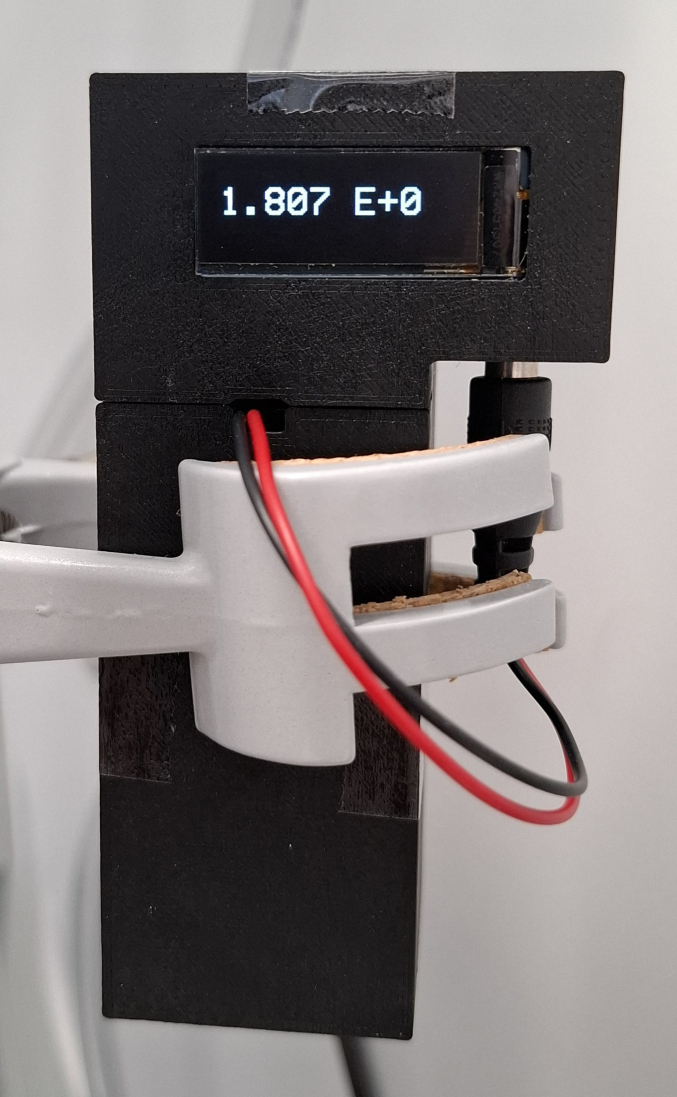
Photo of the application of the device.

It is assumed that the calibration is already done by the builder of the hardware. The switch on the power board should be in position to display the pressure (see Fig. 9).

For everyday use:

• Connect the sensor board to the power board. (done only once)
• Connect the sensor to the apparatus. (done only once)
• Connect the hardware to the power source of choice. The device will start automatically.

## 7 Validation and characterization

**Fig. 10:**
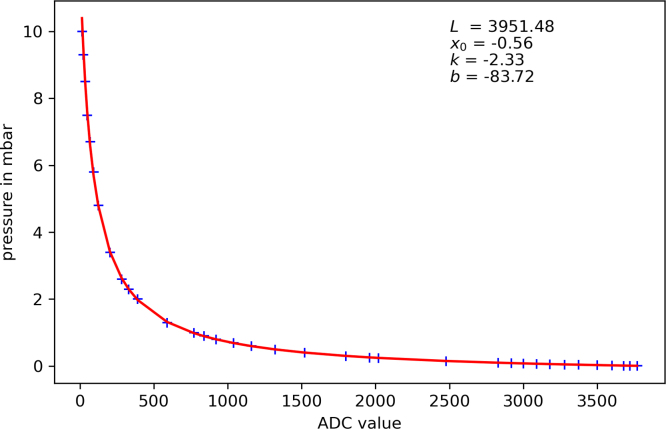
Plot of the individual calibration values and the generated parameters.

Table 2 shows the calibration data for one sensor as an example. With these values, the Python [16] script calculates the calibration parameters (see Fig. 10). It is recommended to have many points at low-pressure values to get a high precision in the lower range.

**Table 2:** Example values for a calibration series.

Pressure (mbar)	ADC values
10	14
9.3	22
8.5	35
7.5	49
6.7	69
5.8	92
4.8	124
3.4	203
2.6	282
2.3	330
2	390
1.3	590
1	770
0.9	840
0.8	920
0.7	1040
0.6	1160
0.5	1320
0.4	1520
0.3	1800
0.255	1960
0.24	2020
0.15	2477
0.1	2830
0.09	2920
0.08	3000
0.07	3090
0.06	3180
0.05	3280
0.04	3373
0.03	3500
0.02	3600
0.013	3680
0.01	3720
0.009	3770

This type of calibration is the “individual calibration” that has to be done for each sensor device produced. As a result of our device manufacturing, we can provide a “standard calibration” with mean values for all parameters of the calibration function. So, an individual calibration is only needed for a higher accuracy. The parameters for the “standard calibration” are:

L=−3842.23667

$$x_{0}=-0.52675$$

k=2.28963

b=3845.51667

The signs of the parameters L and k have to be different. The sign and the magnitude of parameter b depend on the values for k and L. For the calculation of the mean value parameter set for the “standard calibration”. Only values of individual calibration with the same set of signs were used.

Fig. 11 shows a comparison of the two types of calibration (individual and standard) and the deviation of them from the pressure values given by a commercially available device. As can be seen, the individual calibration shows a significantly lower deviation across the entire pressure range compared to the standard calibration. On the upper limit of the pressure range, the deviation reaches its maximum due to the limited range of ADC values (see calibration). A second sensor device was used with the standard calibration to prove a low deviation in the most important pressure range for Schlenk technique from 1 mbar to 1 × 10^-1^ mbar. Although the standard calibration overall shows a larger deviation from the values of the commercially available device, the pressure values are precise enough to provide reliable results when an individual calibration is not possible.

**Fig. 12:**
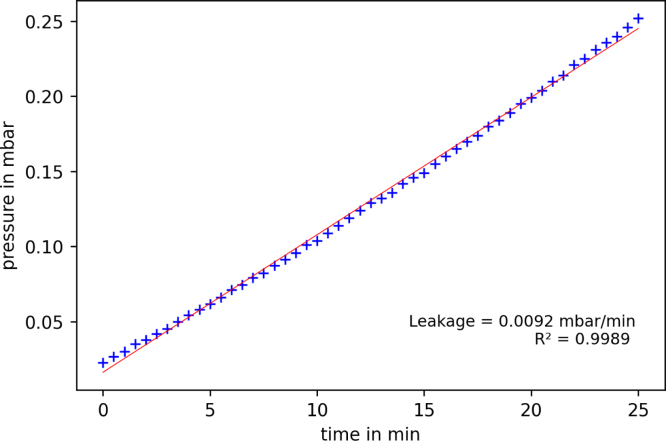
Leakage rate of one vacuum sensor device.

The results of the leakage tests are shown in Fig. 12. Over 25 min the pressure rose from about 2.2 × 10^-2^ mbar to 2.5 × 10^-1^ mbar which results in a leakage rate of 9.2 × 10^-3^ mbar min^−1^. This is a very acceptable leakage rate for the application in Schlenk technique.

## CRediT authorship contribution statement

**Julius Bernd Zimmermann:** Writing – original draft, Visualization, Software, Methodology, Investigation, Formal analysis, Conceptualization. **Marcus Herbig:** Writing – original draft, Supervision.

## Declaration of competing interest

The authors declare that they have no known competing financial interests or personal relationships that could have appeared to influence the work reported in this paper.
